# Prenatal Progestin Exposure-Mediated Oxytocin Suppression Contributes to Social Deficits in Mouse Offspring

**DOI:** 10.3389/fendo.2022.840398

**Published:** 2022-03-15

**Authors:** Saijun Huang, Jiaying Zeng, Ruoyu Sun, Hong Yu, Haimou Zhang, Xi Su, Paul Yao

**Affiliations:** ^1^ Department of Child Healthcare, Affiliated Foshan Maternity & Child Healthcare Hospital, The Second School of Clinical Medicine of Southern Medical University, Foshan, China; ^2^ State Key Lab of Biocatalysis and Enzyme Engineering, School of Life Sciences, Hubei University, Wuhan, China

**Keywords:** autism spectrum disorders, oxytocin, oxidative stress, progestin, social deficits

## Abstract

Epidemiological studies have shown that maternal hormone exposure is associated with autism spectrum disorders (ASD). The hormone oxytocin (OXT) is a central nervous neuropeptide that plays an important role in social behaviors as well as ASD etiology, although the detailed mechanism remains largely unknown. In this study, we aim to investigate the potential role and contribution of OXT to prenatal progestin exposure-mediated mouse offspring. Our *in vitro* study in the hypothalamic neurons that isolated from paraventricular nuclei area of mice showed that transient progestin exposure causes persistent epigenetic changes on the OXT promoter, resulting in dissociation of estrogen receptor β (ERβ) and retinoic acid-related orphan receptor α (RORA) from the OXT promoter with subsequent persistent OXT suppression. Our *in vivo* study showed that prenatal exposure of medroxyprogesterone acetate (MPA) triggers social deficits in mouse offspring; prenatal OXT deficiency in OXT knockdown mouse partly mimics, while postnatal ERβ expression or postnatal OXT peptide injection partly ameliorates, prenatal MPA exposure-mediated social deficits, which include impaired social interaction and social abilities. On the other hand, OXT had no effect on prenatal MPA exposure-mediated anxiety-like behaviors. We conclude that prenatal MPA exposure-mediated oxytocin suppression contributes to social deficits in mouse offspring.

## Introduction

Autism spectrum disorders (ASD) are a series of neurodevelopmental disorders characterized by symptoms including social deficits and restricted or repetitive behaviors (1, 2). While the potential mechanism for ASD remains unclear, many factors, including environmental exposure, sex, and epigenetic modifications, are reported to be associated with ASD development (1, 3, 4). It has been reported that ASD patients have increased steroidogenic activity and that abnormal steroid levels may be involved in ASD development (5, 6). We have previously reported that maternal exposure to either progestin (7, 8) or androgens (9) contribute to autism-like behaviors in offspring; and the epidemiological study shows that maternal hormonal exposure may be associated with autism development (10).

Oral contraceptive hormones, primarily including estrogens and progestins, were originally used starting around 60 years ago for birth control by preventing ovulation; this time period has been reported to coincide with the dramatic increase in ASD prevalence (8, 10). Our epidemiological study has shown that the following 3 risk factors are highly associated with ASD: 1) Use of progestin to prevent threatened abortion, 2) Use of progestin contraceptives at the time of conception, and 3) prenatal consumption of progestin-contaminated food (10). We then hypothesize that maternal exposure to oral contraceptive hormones, especially progestin, may be associated with autism development.

Oxytocin (OXT) is a neuropeptide primarily secreted by hypothalamic neurons that located in either the paraventricular nuclei (PVN) or supraoptic nuclei (SON) (11). OXT, in conjunction with oxytocin receptor (OXTR) (12), has been reported to play an important role in regulation of social recognition and anxiety-like behaviors (13–16) as well as many other kinds of pathophysiological processes (17). OXT/OXTR signaling abnormalities have been associated with ASD (18, 19). We have previously reported that maternal diabetes-mediated OXTR suppression contributes to social deficits in mouse offspring (20), while the detailed mechanism for the role of OXT in ASD development remains largely unknown (21).

Estrogen receptor β (ERβ) is widely expressed in a variety of brain regions and has been reported to be associated with anxiety-like behaviors and ASD development (8, 22–24). We have previously reported that ERβ expression is reduced in the amygdala, contributing to prenatal progestin exposure-mediated autism-like behaviors in rat offspring (7, 8). Additionally, ERβ regulates the expression of superoxide dismutase 2 (SOD2), modulating cellular oxidative stress (25). Interestingly, both ERβ and SOD2 are suppressed in maternal diabetes-mediated autism-like mouse offspring (26). ERβ is highly expressed and co-localized in OXT neurons in the hypothalamic region, and OXT may be regulated directly or indirectly by ERβ, while the possible mechanism remains largely unknown (12, 27, 28).

In this study, we aim to investigate the role and mechanisms for maternal progestin exposure-mediated OXT suppression and its contribution to social behaviors in offspring. Our *in vitro* study in mouse hypothalamic neurons showed that transient treatment by 10µM of medroxyprogesterone acetate (MPA) for 3 days triggers persistent OXT suppression through epigenetic modifications and subsequent dissociation of ERβ and retinoic acid-related orphan receptor α (RORA) (29) from the OXT promoter, indicating that ERβ and RORA may play a role in progestin-mediated OXT suppression. We then conducted the *in vivo* mouse study, and we found that prenatal exposure to MPA triggers OXT suppression as well as autism- and anxiety-like behaviors in offspring. Prenatal OXT deficiency had no effect on prenatal MPA exposure-induced anxiety-like behavior, but it partly mimicked prenatal MPA exposure-mediated social deficits in offspring. We next conducted postnatal gene manipulation of ERβ and RORA targeting to hypothalamic OXT neuron-located PVN area, and we found that postnatal ERβ expression partly ameliorated prenatal MPA exposure-induced social deficits, while postnatal RORA expression had no effect. Furthermore, postnatal OXT peptide injection to the third ventricle partly ameliorated prenatal MPA exposure-induced social deficits in offspring as well. We conclude that prenatal MPA exposure-mediated oxytocin suppression contributes to social deficits in mouse offspring.

## Materials and Methods

An expanded section for Materials and Methods is available in **Supplementary Information** (see **Data S1**), and the details for used primers are available in **Table S1**.

### Reagents and Materials

The primary hypothalamus neurons were isolated from the paraventricular nucleus (PVN) area of experimental mice. The antibodies for β-actin (sc-47778), p53 (sc-126), RORA (sc-518081), RXRα (sc-515929) and SOD2 (sc-30080) were purchased from Santa Cruz Biotechnology. The oxytocin (OXT) from tissue, culture medium, serum and cerebrospinal fluid (CSF) was determined using the Oxytocin ELISA Kit (ab133050) according to manufacturers’ instructions.

### Generation of OXT Reporter Construct

The genomic DNA was purified from primary mouse hypothalamic neurons, and the mouse OXT promoter (2kb upstream + first exon) was identified from Ensembl gene ID: OXT-201 ENSMUST00000028764.6, and amplified by PCR, then subcloned into the pGL3-basic vector (# E1751, Promega) using the following primers with underlined restriction sites: OXT forward: 5’-gcgc-acgcgt- cta acc taa agc cca aag ctg -3’ (Mlu I) and OXT reverse: 5’- gtac- aagctt- ctt gcg cat atc cag gtc cag -3’ (Hind III). To map the progestin-responsive element on the OXT promoter, the related OXT deletion reporter constructs were generated using PCR techniques and subcloned into pGL3-basic vector (30).

### Generation of Expression Lentivirus

The mouse ERβ expression lentivirus was prepared previously in our lab (20). The cDNA for mouse RORA was purchased from Open Biosystems and then amplified using the following primers with underlined restriction sites: RORA forward primer: 5’- gtac - gggccc- atg gag tca gct ccg gca gcc -3’ (ApaI) and RORA reverse primer: 5’- gtac - tctaga- tta ccc atc gat ttg cat ggc -3’ (Xba1), and then subcloned into the pLVX-Puro vector (from Clontech). The lentivirus for ERβ, RORA, and empty control were expressed using Lenti-X™ Lentiviral Expression Systems (from Clontech) and concentrated according to manufacturers’ instructions (26).

### DNA Methylation Analysis

The DNA methylation on the OXT promoter was evaluated using a methylation-specific PCR-based method as described previously with minor modifications (31–33). The mouse genomic DNA was extracted and purified from primary hypothalamic neurons, and then treated by bisulfite modification through EpiJET Bisulfite Conversion Kit (#K1461, from Fisher). The treated DNA was then amplified using the following primers: Methylated primer: forward 5’- tga aaa ata gtt ttt ggt tag ggc -3’ and reverse 5’- ctc tta aat caa att att cca cgc t -3’; Unmethylated primer: forward 5’- gaa aaa tag ttt ttg gtt agg gtg t -3’ and reverse 5’- ctc tta aat caa att att cca cac t -3’. Product size: 198bp (methylated) & 197bp (unmethylated); CpG island size: 227bp; Tm: 68.4°C. The final DNA methylation results were normalized by DNA unmethylated results as input.

### *In Vivo* Mouse Experiments

Generation of neuron-specific OXT knockout mice. The OXT^fl/fl^ mouse, which has loxP flanking sites targeting exon 3 of the OXT gene, was generated by *in vitro* fertilization and was obtained for this study as a generous gift from Dr. Haimou Zhang (Hubei University). The Oxytocin-Ires Cre mice (Oxt^Cre^, #024234), which expresses Cre recombinase under the control of the oxytocin promoter, was obtained from Jackson Laboratories. To generate neuron-specific OXT^-/-^ null mice (Oxt^Cre^-OXT^fl/fl^), OXT^fl/fl^ mice were cross-bred with Oxt^Cre^ mice for over 4 generations on the C57BL/6J background. Positive offspring were confirmed by genotyping through PCR using specific primers (see **Table S1**) for the presence of both loxP sites within OXT alleles and Cre recombinase (34, 35). The experimental animals were either OXT wild type (WT) or OXT null (OXT^-/-^) mice with C57BL/6J genetic background as described above.

Mouse Protocol 1: Prenatal treatment by progestin MPA or OXT deficiency. Female mice (3-month old) were mated with males, and the pregnant dams were verified, then received either MPA treatment (20 mg/kg body weight, which is similar or equal to high-dose of women exposure) or control (CTL) group that received vehicle only, which containing 1% ethanol in organic sesame oil, and 0.1 ml of drugs were given every 2 days by peritoneal injection from day 1 until offspring delivery for ~21 days in total. The above treated dams were then randomly assigned to the below 4 groups: Group 1: OXT WT background dams receiving CTL injection (CTL/WT); Group 2: OXT WT background dams receiving MPA injection (MPA/WT); Group 3: OXT null background dams receiving CTL injection (CTL/OXT^-/-^); Group 4: OXT null background dams receiving MPA injection (MPA/OXT^-/-^). 10 dams were assigned for each group, and one representative offspring was selected randomly from each dam for experiments and analysis. Nine representative offspring were selected from the 10 in total in order to account for potential death of an experimental animal during the process. Hypothalamic neurons from PVN area were isolated on embryonic day 18 (E18), and the offspring were then fed by normal chow until 7-8 weeks old, after which they were given behavior tests. The offspring were then sacrificed; the serum and CSF were collected for OXT analysis and various brain tissues, including the amygdala, hypothalamus (PVN area) and hippocampus, were isolated for further biological assays, including gene expression and oxidative stress.

Mouse Protocol 2: Postnatal manipulation of ERβ/RORA lentivirus-carried expression. At 6-week of age, offspring of OXT wild type background that received either the CTL or MPA treatment as described in Mouse Protocol 1 were anesthetized by a mixture of ketamine (90 mg/kg) and xylazine (2.7 mg/kg) and implanted with a guide cannula targeting the PVN area by the direction of an ultra-precise stereotax (Kopf Instruments) using the coordinates of 0.85 mm posterior to the bregma, 0.15 mm lateral to the midline, and 4.8 mm below the skull surface (36). The lentivirus for expression of ERβ (↑ERβ), RORA (↑RORA), or empty (EMP) was infused immediately by a flow rate of 0.5 µl/h after placement of the cannula and minipump, and in total, 0.5μl of (2×10^3^ cfu) lentivirus was infused in 1 hour, and the lentivirus was dissolved in artificial cerebrospinal fluid (aCSF), which containing 140 mM NaCl, 3 mM KCl, 1.2 mM Na2HPO4, 1 mM MgCl2, 0.27 mM NaH2PO4, 1.2 mMCaCl2, and 7.2 mM dextrose in pH 7.4. The experimental animals were randomly separated into the following 4 groups (10 mice each group). Group 1: CTL treated offspring received vehicle lentivirus infusion (CTL/P-EMP); Group 2: MPA treated offspring received vehicle lentivirus infusion (MPA/P-EMP); Group 3: MPA treated offspring received ERβ lentivirus infusion (MPA/P-↑ERβ); Group 4: MPA treated offspring received RORA lentivirus infusion (MPA/P-↑RORA). To confirm a successful lentivirus injection into PVN area, the cannula placement was checked histologically postmortem by injection of 0.5μl India ink. Animals whose dye injections were not located in the PVN area were excluded from the final analysis, and the offspring were used for behavior tests after two-week of lentivirus infusion followed with biological assays as indicated in Mouse Protocol 1 (37).

Mouse Protocol 3: Postnatal administration of OXT peptides. The offspring (6-week old) from Mouse Protocol 1 were anesthetized and implanted with a guide cannula targeting the third ventricle at the midline coordinates of 1.8 mm posterior to the bregma and 5.0 mm below the skull surface (36). Two weeks were allowed for mice to recover from surgery, and each mouse then received injection with either aCSF as vehicle (VEH) control or oxytocin peptide (OXT, dissolved in aCSF) *via* pre-implanted cannula (36, 38). The experimental animals were then randomly separated into the following 4 groups (10 mice each group). Group 1: CTL treated offspring received vehicle injection (CTL/P-VEH); Group 2: MPA treated offspring received vehicle injection (MPA/P-VEH); Group 3: CTL treated offspring received OXT peptide injection (MPA/P-OXT); Group 4: MPA treated offspring received OXT peptide injection (MPA/P-OXT). The oxytocin (0.1 mM, diluted in aCSF, 1 μg/20μl aCSF) or vehicle was locally administered *via* the installed catheter (39). 20 min (including a period for 5 min-adaptation in the test cage) after the injection, the offspring were used for behavior tests followed by biological assays, as indicated in Mouse Protocol 1 (37).

### Animal Behavior Tests

The animal behavior tests were evaluated at ages of 7-8 weeks old from offspring unless otherwise mentioned. Anxiety-like behavior was determined by the marble-burying test (MBT) and the elevated plus maze (EPM) tests (7). Autism-like behavior was determined by ultrasonic vocalization (USV), social interaction (SI) test and a three-chambered social test (40–42), and the details for these tests are described in **Supplementary Information**.

### Isolation of Brain Tissues

The brain tissues were isolated from experimental offspring for further biological assays. The experimental mouse was deeply anesthetized through free breathing of isoflurane vapor (> 5%). The whole blood was then withdrawn by heart puncture for PBMC isolation and the mouse was perfused transcardially by 20 ml cold perfusion solution for 5 min. The skull was cut using a pair of small surgical scissors and the brain was carefully freed from the skull before being transferred to a petri dish (60 mm×15 mm) that was filled with ice-cold DPBS solution. The targeted brain regions, including the amygdala, hypothalamus (PVN area) and hippocampus, were dissected under the surgical microscope under the referred location from the atlas outlined in *The Mouse Brain in Stereotaxic Coordinates (3rd Edition)*. A separate petri dish was prepared for each of the target regions. The whole dissection process was carried out in the span of no more than one hour. The dissected tissues were then frozen at -80°C for either immediate use or later biological assays (43, 44).

### Collection of Cerebrospinal Fluid

The procedure for CSF collection is based on a previously established protocol with minor modifications. In brief, the mouse was anesthetized and the shaved head was clamped in place for dissection under a dissecting microscope. The layers of muscles were carefully dissected away using forceps and the dura over the cisterna magna was exposed. This area has large blood vessels running through, which is optimal for capillary insertion and CSF collection. The angle of the glass capillary was carefully adjusted and the sharpened tip of glass capillary was aligned and eventually tapped through the dura to collect CSF using a micromanipulator control. Approximately 20 µl of CSF was automatically drawn into the capillary tube once the opening was punctured. The glass capillary was gently removed from the mouse by micromanipulator control and the CSF was then mixed with 1 µl of 20x protease inhibitor in a 1.5 ml centrifuge tube for a quick centrifugation (pulse spin for 5 seconds at maximal speed), and the CSF samples were aliquoted for either immediate analysis or stored at -80°C (45).

### *In Vitro* Primary Culture of Hypothalamic Neurons

The isolation of hypothalamic neurons was carried out following a previously described procedure with minor modifications. Three to five hypothalami from PVN area of mice on embryonic day 18 (E18 rats) were isolated, pooled, and then dissociated into single cell suspension by trituration. They were then transferred to a culture dish, which containing primary DMEM culture medium, 10% FBS, 10% heat-inactivated horse serum, 20mM D-glucose and combined antibiotics (from Invitrogen). The osmolarity of medium was then adjusted to 320-325 mOsm using glucose. The subsequent cell suspension was then split into tissue culture flasks that coated with 100μg/ml of poly-L-lysine (Sigma). 24 hours of incubation were allowed for cells to attach to the flask at 37°C with 5% CO2, the medium was then refreshed for cells to growth until confluent for further biological assays (46). The isolated primary hypothalamic neurons were used for *in vitro* cell culture study until passage 3. For mapping of progestin-responsive element on the OXT promoter, the cells were immortalized by an hTERT lentivirus vector for a longer life span (up to passage 12) to achieve better transfection efficiency and higher experimental stability as described previously (47, 48).

## Results

### Transient Progestin Treatment Causes Persistent OXT Suppression and Oxidative Stress; ERβ Expression Completely, While RORA Expression Partly, Reverses This Effect

**Figure 1:**
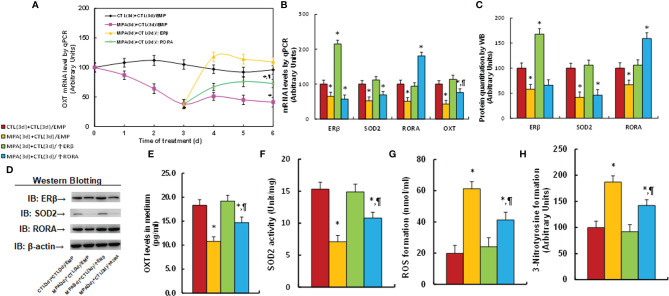
Transient MPA treatment causes persistent OXT suppression and oxidative stress; ERβ expression completely, while RORA expression partly, reverses this effect. Mouse hypothalamic neurons were treated with either 10 μM MPA or vehicle control (CTL) for 3 days. The cells were infected by empty (CTL), ERβ (↑ERβ), or RORA (↑RORA) lentivirus on day 3 and the cells were then cultured without MPA for another 3 days in the presence of 1% FBS before being harvested for biological assays. **(A)** OXT mRNA levels on different time points, n=4, *P < 0.05, vs. day 0 group; ^¶^ *P* < 0.05, vs. day 3 group. (B–H) Biological assays on day 6. **(B)** mRNA levels, n=4. **(C)** Protein quantitation, n=5. **(D)** Representative western blots for **(C)**. **(E)** OXT levels in culture medium, n=5. **(F)** SOD2 activity, n=5. **(G)** ROS generation, n=5. **(H)** 3-nitrotyrosine generation, n=5. *P < 0.05, vs. CTL(3d)+CTL(3d)/EMP group; ^¶^ *P* < 0.05, vs. MPA(3d)+CTL(3d)/EMP group. Data were expressed as mean ± SD.

We first determined the possible effect of MPA treatment on OXT expression. Mouse hypothalamic neurons were treated by MPA for 3 days and then cultured for another 3 days in the absence of MPA, but with the infection of either ERβ (↑ERβ) or RORA lentivirus (↑RORA) for biological assays. Our results showed that 3-day MPA treatment significantly suppressed OXT mRNA levels and that OXT mRNA remained low after removal of MPA. Infection of ERβ lentivirus completely, while RORA expression partly, reversed this effect (see **Figures 1A, B**). We also measured mRNA expression of these genes at the end of the treatment on day 6, and the results showed that lentivirus infection of either ERβ or RORA was successful. Transient MPA treatment significantly suppressed expression of ERβ, SOD2 and RORA, and the expression remained low during subsequent MPA absence (see **Figure 1B**). We then evaluated protein levels of these genes by either western blotting (see **Figures 1C, D**, **S1A**) or ELISA for OXT (see **Figure 1E**), and the expression pattern was similar to that of mRNA levels. In addition, we conduct immunostaining of OXT for the hypothalamic neurons that isolated from PVN area of mice, and the results showed that almost all the neurons had OXT expression (see **Figure S2**), indicating a successful OXT neuron preparation. We also evaluated the potential effect of MPA on OXTR expression and the results showed that MPA had no effect, while ERβ expression significantly increased OXTR mRNA levels (see **Figure S3**). We then measured the effect of MPA on oxidative stress, and the results showed that MPA treatment significantly decreased SOD2 activity (see **Figure 1F**) and increased ROS formation (see **Figure 1G**) and 3-nitrotyrosine formation (see **Figure 1H**). Again, ERβ expression completely, while RORA expression partly, reversed this effect. Furthermore, we determined the potential effect of other progestins on OXT expression and epigenetic changes. The results showed that estrogen (E2), progesterone (P2) and NGM had no significant effect, while almost all transient treatments of progestin, including LNG, NES, NET, NETA, NEN and OHPC, induced persistent OXT suppression and increased H3K27me2 modification on the OXT promoter (see **Table 1**). We conclude that transient progestin treatment causes persistent OXT suppression and oxidative stress in hypothalamic neurons.

**Table 1:** Transient progestin exposure causes persistent epigenetic changes on the OXT promoter and the subsequent OXT suppression.

Transient Progestin Exposure	OXT mRNA level by Qpcr (% control)	H3K27me2 modification by ChIP (% control)
E2	106 ± 11	91 ± 12
P4	92 ± 10	108 ± 11
LNG	76 ± 9*	163 ± 8*
MPA	43 ± 10*	215 ± 11*
NES	69 ± 12*	121 ± 10
NET	58 ± 11*	147 ± 10*
NETA	71 ± 8*	168 ± 12*
NEN	62 ± 10*	177 ± 9*
NGM	87 ± 11	119 ± 13
OHPC	64 ± 12*	188 ± 12*

Primary mouse hypothalamic neurons were treated with either 10 μM of progestin (dissolved in 0.1% DMSO) or vehicle control for 3 days before then being cultured for another 3 days in the absence of progestin in the presence of 1% FBS during 6-day treatment. The cells were harvested for mRNA analysis and ChIP analysis on the OXT promoter. E2, 17β-estradiol; P4, progesterone; LNG, levonorgestrel; MPA, medroxyprogesterone acetate; NES, nestorone; NET, norethindrone; NETA, norethindrone acetate; NEN, norethynodrel; NGM, norgestimate; OHPC, hydroxyprogesterone caproate; *, P<0.05, vs control group. n=4, results were expressed as mean ± SD.

### MPA Induces OXT Suppression by Epigenetic Modifications and Subsequent Dissociation of ERβ and RORA From the OXT Promoter

**Figure 2:**
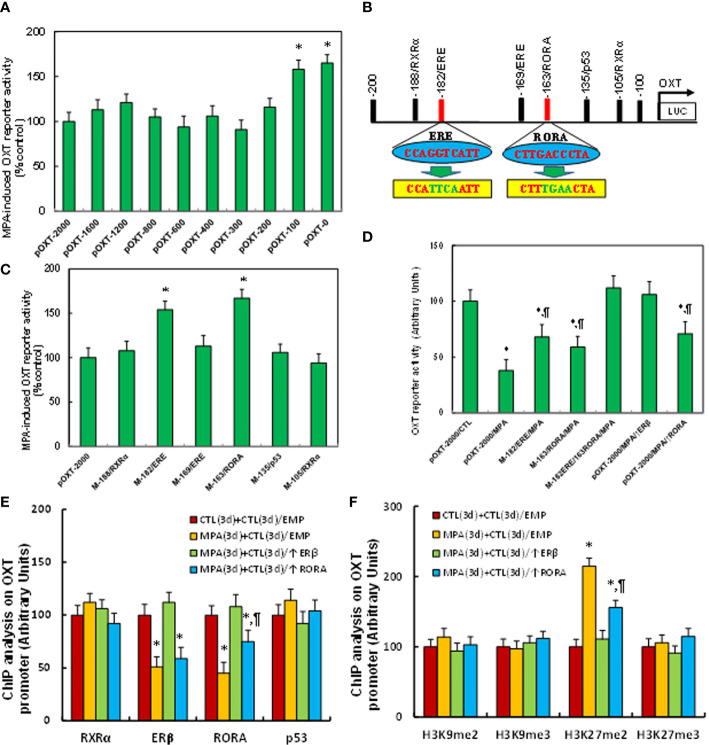
MPA induces OXT suppression by epigenetic modifications and the subsequent dissociation of ERβ and RORA from the OXT promoter. **(A)** The immortalized mouse hypothalamic neurons from PVN area were transfected by either OXT full length (pOXT-2000) or deletion reporter constructs. After 24 hours, cells were then treated by either control or 10μM MPA for 3 days and the OXT reporter activities were then calculated, n=5. *P < 0.05, vs. pOXT-2000 group. **(B)** Schematic model for the possible transcriptional binding element on the OXT promoter with one of ERE and RORA binding site (in red) as well as related mutation sites (in green). **(C)** The cells were transfected with either wild type OXT reporter construct (pOXT-2000) or single point mutation construct as shown in **(B)** and then treated by either control or MPA for 3 days, and the OXT reporter activities were then determined, n=5. *P < 0.05, vs. pOXT-2000 group. **(D)** The cells were transfected by either OXT full length (pOXT-2000), single mutant, double mutants as indicated, or infected by ERβ lentivirus (↑ERβ), and then treated by either control or MPA for 3 days, and the OXT reporter activities were then determined, n=5. *P < 0.05, vs. pOXT-2000/CTL group; ^¶^ *P* < 0.05, vs. pOXT-2000/MPA group. **(E)** ChIP analysis for transcription factor binding ability assay, n=4. **(F)** ChIP analysis for histone 3 methylation, n=4. *P < 0.05, vs. CTL(3d)+CTL(3d)/EMP group; ^¶^ *P* < 0.05, vs. MPA(3d)+CTL(3d)/EMP group. Data were expressed as mean ± SD.

We evaluated the potential molecular mechanism for MPA-induced OXT suppression. The conditionally immortalized hypothalamic neurons from PVN area were transfected by either OXT full length (pOXT-2000) or deletion reporter constructs and then treated by MPA for luciferase reporter assay. Our results showed that MPA-induced OXT suppression had no significant changes in the constructs of -2000, -1600, -1200, -800, -600, -400 and -200, while the suppression was significantly diminished in deletion constructs of -100 and -0, indicating that the MPA-responsive element is located in the range of -200~-100 on the OXT promoter (see **Figure 2A**). We then searched all the potential binding motifs in the range of -200~-100 on the OXT promoter and found that there were two RXRα motifs at -188 and -105, two estrogen response element (ERE) motifs at -182 (marked in red) and -169, one motif for RORA at -163 (marked in red) and one for p53 at -135, respectively (see **Figure 2B**). We then mutated these potential binding motifs respectively in the OXT full length reporter constructs and transfected them for reporter assay. The results showed that single mutants (marked in green, see **Figure 2B**) of ERE at -162 (M-182/ERE) and RORA at -163 (M-163/RORA) significantly diminished MPA-induced OXT suppression, while other single mutants had no effect (see **Figure 2C**). We then transfected either single or double mutants of M-182/ERE and M163/RORA to investigate the effect of MPA, and the result showed that single mutant of either M-182/ERE or M-163/RORA partly, while double mutant M-182/ERE/163/RORA completely, reversed MPA-induced suppression. ERβ expression completely, but RORA expression partly, reversed MPA-induced suppression (see **Figure 2D**). We also evaluated the binding ability of these motifs by ChIP techniques, and the results showed that MPA treatment significantly decreased the binding abilities of ERβ and RORA on the OXT promoter. Again, ERβ expression completely, but RORA expression partly, reversed MPA-induced suppression (see **Figure 2E**). We finally evaluated MPA-mediated epigenetic changes on the OXT promoter by ChIP techniques. The results showed that MPA treatment significantly increased H3K27me2 modifications on the OXT promoter, but had no effect on H3K9me2, H3K9me3 or H3K27me3. ERβ expression completely, while RORA expression partly, reversed this effect (see **Figure 2F**). In addition, we found that MPA treatment had no effect on the OXT promoter for DNA methylation (see **Figure S4**), histone 4 methylation (see **Figure S5A**) and histone 3 acetylation (see **Figure S5B**). We conclude that MPA induces OXT suppression by epigenetic modifications and the subsequent dissociation of ERβ and RORA from the OXT promoter.

### Prenatal OXT Deficiency Mimics Prenatal MPA Exposure-Mediated OXT Suppression and Oxidative Stress

**Figure 3:**
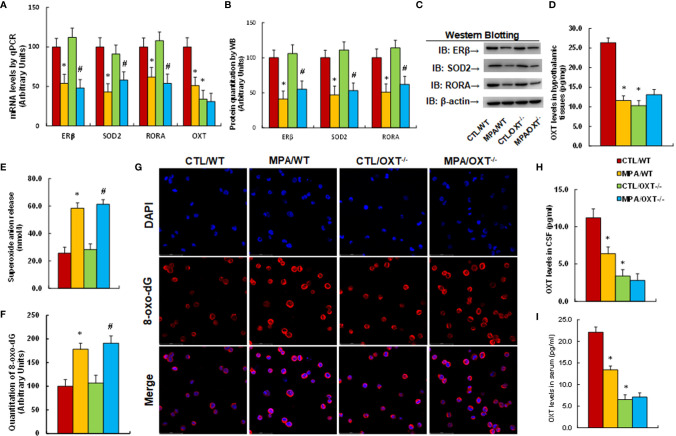
Prenatal OXT deficiency mimics prenatal MPA exposure-mediated OXT suppression and oxidative stress. The OXT wild type (WT) or OXT null (OXT^-/-^) background dams were treated by either control (CTL) or MPA and the hypothalamic neurons or tissues from PVN area of offspring were isolated for biological assays. **(A-D)** The hypothalamic tissues were isolated for analysis. **(A)** mRNA levels, n=4. **(B)** Protein quantitation, n=5. **(C)** Representative western blots for **(B)**. **(D)** OXT levels in hypothalamic tissues, n=5. **(E)** Superoxide anion release in hypothalamic tissues, n=5. **(F, G)** Immunostaining of hypothalamic neurons. **(F)** 8-oxo-dG staining quantitation, n=5. **(G)** Representative 8-oxo-dG staining (red) and DAPI staining (blue). **(H)** OXT levels in CSF, n=5. **(I)** OXT levels in serum, n=5. *P < 0.05, vs. CTL/WT group; ^#^ *P* < 0.05, vs. CTL/OXT-/- group. Data were expressed as mean ± SD.

We determined the effect of prenatal OXT deficiency on prenatal MPA exposure-mediated OXT suppression and oxidative stress. The OXT wild type (WT) or OXT null (OXT^-/-^) background dams were exposed to either control (CTL) or MPA and the hypothalamic neurons or tissues from PVN area of offspring were isolated for analysis. We first evaluated gene expression in hypothalamic tissues, and found that MPA exposure significantly decreased mRNA levels of ERβ, SOD2, RORA and OXT in hypothalamic tissues. Prenatal OXT deficiency showed no further effect, although it decreased OXT mRNA levels in the control (CTL) group (CTL/OXT-/-), indicating that OXT knockdown in these animals was successful (see **Figure 3A**). We also measured protein levels for the genes through either western blotting (see **Figures 3B, C**, **S1B**) or ELISA for OXT (see **Figure 3D**), and the expression pattern was similar to that of mRNA levels. In addition, we measured gene expression in tissues of both the amygdala (see **Figure S6A**) and hippocampus (see **Figure S6B**), and the results showed that MPA exposure decreased mRNA levels of ERβ, SOD2 and RORA in the amygdala but had no effect in the hippocampus. OXT knockdown showed no further effect. We also evaluated the effect of MPA and OXT deficiency on oxidative stress in hypothalamic tissues, and the results showed that prenatal MPA exposure significantly increased superoxide anion release (see **Figure 3E**) and 8-oxo-dG formation (see **Figures 3F, G**), while prenatal OXT deficiency showed no effect. We then evaluated OXT peptide levels in both the CSF (see **Figure 3H**) and serum (see **Figure 3I**), and found that prenatal MPA exposure significantly decreased OXT levels, and prenatal OXT deficiency achieved a further decrease. We conclude that prenatal OXT deficiency mimics prenatal MPA exposure-mediated OXT suppression and oxidative stress.

### Prenatal OXT Deficiency Partly Mimics Prenatal MPA Exposure-Mediated Social Deficits in Mouse Offspring

**Figure 4:**
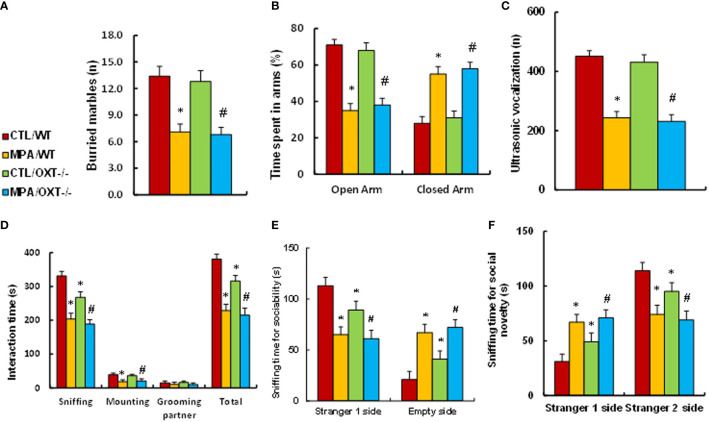
Prenatal OXT deficiency partly mimics prenatal MPA exposure-mediated social deficits in mouse offspring. The OXT wild type (WT) or OXT null (OXT^-/-^) background dams were treated by either control (CTL) or MPA, and the subsequent offspring were used for animal behavior tests. **(A)** MBT test, n=9. **(B)** EPM test, n=9. **(C)** Ultrasonic vocalization, n=9. **(D)** Social interaction (SI) test, n=9. **(E, F)** Three-chambered social tests for sociability **(E)** and social novelty **(F)**, n=9. *P < 0.05, vs. CTL/WT group; ^#^ *P* < 0.05, vs. CTL/OXT-/- group. Data were expressed as mean ± SD.

We determined the potential effect of prenatal MPA exposure and OXT deficiency on animal behaviors. We first evaluated anxiety-like behaviors, and our results showed that offspring in the prenatal MPA exposure (MPA/WT) group buried less marbles in the marble-burying test (MBT) test (see **Figure 4A**) and spent less time in the Open Arm and more time in Closed Arm during the elevated plus maze (EPM) test (see **Figure 4B**) compared to the control (CTL/WT) group. We then evaluated autism-like behaviors, and the results showed that mice in the MPA/WT group had fewer ultrasonic vocalizations in the USV tests (see **Figure 4C**) and spent significantly less time sniffing, mounting and interacting in total during the social interaction (SI) tests (see **Figure 4D**). They spent less time sniffing in the Stranger 1 side and more time in the Empty side for sociability (see **Figure 4E**); additionally, they spent more time in the Stranger 1 side and less time in the Stranger 2 side for social novelty (see **Figure 4F**) during the three-chambered social test compared to the CTL/WT group. OXT deficiency had no effect on the MBT, EPM or USV tests, while it slightly decreased sniffing and total interaction time in the SI test and slightly decreased social ability and social novelty in the three-chambered social tests. We conclude that prenatal OXT deficiency partly mimics prenatal MPA exposure-mediated social deficits in mouse offspring.

### Postnatal ERβ Expression Completely, While Postnatal RORA Expression Partly, Reverses Prenatal MPA Exposure-Mediated OXT Suppression and Oxidative Stress in Offspring

**Figure 5:**
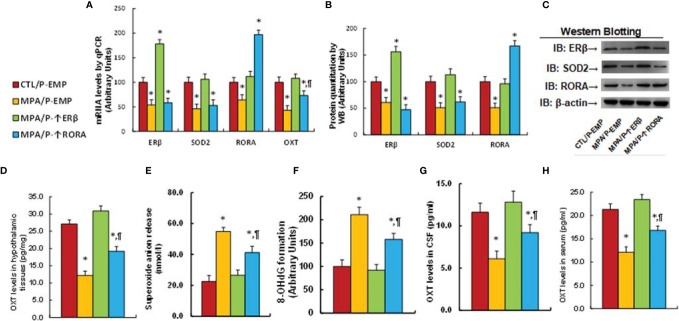
Postnatal ERβ expression completely, while postnatal RORA expression partly, reverses prenatal MPA exposure-mediated OXT suppression and oxidative stress in offspring. The pregnant dams were treated with either control (CTL) or MPA, and the subsequent offspring received either empty (EMP), ERβ (↑ERβ) or RORA (↑RORA) lentivirus, and the offspring were then sacrificed for biological assays. (A–F) The hypothalamic tissues from PVN area were isolated for biological assays: **(A)** mRNA levels, n=4. **(B)** Protein quantitation, n=5. **(C)** Representative western blots for **(B)**. **(D)** OXT levels in hypothalamic tissues, n=5. **(E)** Superoxide anion release, n=5. **(F)** 8-OHdG generation, n=5. **(G)** OXT levels in CSF, n=5. **(H)** OXT levels in serum, n=5. *P < 0.05, vs. CTL/P-EMP group; ^¶^ *P* < 0.05, vs. MPA/P-EMP group. Data were expressed as mean ± SD.

Pregnant dams were given either control (CTL) or MPA treatment, and the subsequent offspring received either empty (EMP), ERβ (↑ERβ) or RORA (↑RORA) lentivirus in the PVN area before then being sacrificed for analysis. We first determined gene expression in the hypothalamic tissues that isolated from PVN area of offspring, and found that infection of either ERβ or RORA lentivirus significantly increased mRNA levels, respectively, indicating a successful gene manipulation. Additionally, ERβ expression (MPA/P-↑ERβ) completely reversed MPA exposure-mediated gene suppression of ERβ, SOD2, RORA and OXT. RORA expression (MPA/P-↑RORA) showed no effect on ERβ and SOD2, while it partly reversed MPA exposure-mediated OXT suppression (see **Figure 5A**). We also measured protein levels for the genes using either western blotting (see **Figures 5B, C**, **S1C**) or ELISA for OXT (see **Figure 5D**), and the expression pattern was similar to that of mRNA levels. Moreover, we measured gene expression in the other brain regions, and the results showed that ERβ expression completely reversed MPA exposure-mediated gene suppression of ERβ, SOD2 and RORA in the amygdala, while RORA expression showed no effect (see **Figure S7A**). Neither prenatal MPA exposure nor postnatal gene manipulation showed any effect on gene expression in the hippocampus (see **Figure S7B**). We also evaluated the effect of MPA exposure and postnatal gene manipulation on oxidative stress in hypothalamic tissues, and the results showed that postnatal ERβ expression completely, while RORA expression partly, reversed prenatal MPA exposure-mediated increased superoxide anion release (see **Figure 5E**) and 8-OHdG formation (see **Figure 5F**). We then evaluated OXT peptide levels in both the CSF (see **Figure 5G**) and serum (see **Figure 5H**), and the results showed that postnatal ERβ expression completely, while RORA expression partly, reversed prenatal MPA exposure-mediated OXT suppression. We conclude that postnatal ERβ expression completely, while postnatal RORA expression partly, reverses prenatal MPA exposure-mediated OXT suppression and oxidative stress in offspring.

### Postnatal ERβ Expression Partly Ameliorates Prenatal MPA Exposure-Mediated Social Deficits in Mouse Offspring, While Postnatal RORA Expression Has no Effect

**Figure 6:**
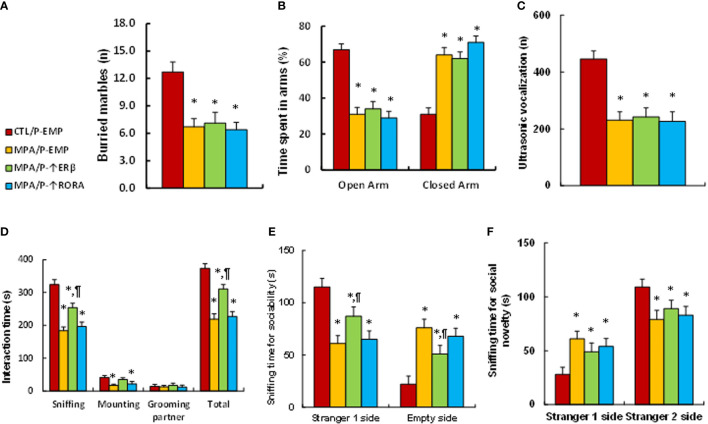
Postnatal ERβ expression partly ameliorates prenatal MPA exposure-mediated social deficits in mouse offspring, while postnatal RORA expression has no effect. The pregnant dams were treated with either control (CTL) or MPA, and the subsequent offspring received empty (EMP), ERβ (↑ERβ) or RORA (↑RORA) lentivirus before then being used for animal behavior tests. **(A)** MBT tests, n=9. **(B)** EPM tests, n=9. **(C)** Ultrasonic vocalization, n=9. **(D)** Social interaction (SI) test, n=9. **(E, F)** Three-chambered social tests for sociability **(E)** and social novelty **(F)**, n=9. *P < 0.05, vs. CTL/WT group; ^#^ *P* < 0.05, vs. CTL/OXT-/- group. Data were expressed as mean ± SD.

We evaluated animal behaviors of offspring with prenatal MPA exposure and postnatal gene manipulation. Our results showed that postnatal expression of either ERβ or RORA showed no effect on MPA exposure-mediated anxiety-like behaviors, as measured using the marble-burying test (MBT) test (see **Figure 6A**) and elevated plus maze (EPM) test (see **Figure 6B**). We also evaluated autism-like behaviors, and the results showed that postnatal expression of either ERβ or RORA showed no effect on MPA exposure-mediated decreased ultrasonic vocalization in USV tests (see **Figure 5C**). On the other hand, postnatal ERβ expression partly ameliorated MPA exposure-mediated impaired social interaction, including sniffing and total interaction time, as measured in the social interaction (SI) tests (see **Figure 6D**). Additionally, it partly ameliorated MPA exposure-mediated impaired sociability (see **Figure 6E**) but not social novelty (see **Figure 6F**) during the three-chambered social test. Postnatal RORA expression showed no effect on MPA exposure-mediated behaviors in offspring (see **Figures 6C**–**F**). We conclude that postnatal ERβ expression partly ameliorates prenatal MPA exposure-mediated social deficits in mouse offspring.

### Postnatal Injection of OXT Peptide Partly Reverses Prenatal MPA Exposure- Mediated Social Deficits in Mouse Offspring

**Figure 7:**
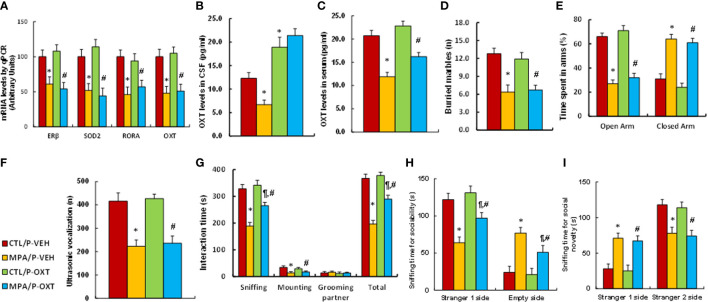
Postnatal injection of OXT partly ameliorates prenatal MPA exposure-mediated social deficits in mouse offspring. The pregnant dams were treated with either control (CTL) or MPA, and the subsequent offspring received either vehicle (VEH) or OXT peptide injection through the third ventricle. The animals were then used for analysis. **(A)** The hypothalamic tissues were used for mRNA analysis, n=4. **(B)** OXT levels in CSF, n=5. **(C)** OXT levels in serum, n=5. **(D–I)** Offspring were used for animal behavior tests. **(D)** MBT tests, n=9. **(E)** EPM tests, n=9. **(F)** Ultrasonic vocalization, n=9. **(G)** Social interaction (SI) test, n=9. **(H, I)** Three-chambered social tests for sociability **(H)** and social novelty **(I)**, n=9. *P < 0.05, vs. CTL/P-VEH group; ^¶^ *P* < 0.05, vs. MPA/P-VEH group; ^#^ *P* < 0.05, vs. CTL/P-OXT group. Data were expressed as mean ± SD.

Pregnant dams were treated with either control (CTL) or MPA, and the subsequent offspring received either vehicle (VEH) or OXT peptide injection through the third ventricle for biological assays. We first determined gene expression in hypothalamic tissues that isolated from PVN area, and found that OXT peptide showed no effect on MPA exposure-mediated gene suppression of ERβ, SOD2, RORA or OXT (see **Figure 7A**). We also measured OXT peptide levels after OXT injection, and found that postnatal OXT injection significantly increased OXT levels in the CSF compared to the control (CTL/P-VEH) group (see **Figure 7B**) and also partly reversed MPA exposure-mediated decreased OXT serum levels (see **Figure 7C**). We evaluated animal behaviors in the offspring, and our results showed that postnatal OXT injection showed no effect on MPA exposure-mediated anxiety-like behaviors, as measured through the marble-burying test (MBT) test (see **Figure 7D**) and elevated plus maze (EPM) test (see **Figure 7E**). We also evaluated autism-like behaviors, and the results showed that postnatal OXT injection showed no effect on MPA exposure-mediated decreased ultrasonic vocalization in USV tests (see **Figure 7F**). On the other hand, postnatal OXT injection partly ameliorated MPA exposure-mediated impaired social interaction, as indicated through sniffing and total interaction time during the social interaction (SI) tests (see **Figure 7G**). Additionally, it partly ameliorated MPA exposure-mediated impaired sociability (see **Figure 7H**) but not social novelty (see **Figure 7I**) during the three-chambered social test. We conclude that postnatal OXT injection partly ameliorates prenatal MPA exposure-mediated social deficits in mouse offspring.

## Discussion

In this study, we found that transient progestin treatment triggers persistent epigenetic changes and OXT suppression in hypothalamic neurons. Prenatal MPA exposure induces OXT suppression, oxidative stress and social deficits in offspring. OXT knockdown mice partly mimics, while postnatal ERβ expression or postnatal OXT peptide injection partly ameliorates, prenatal MPA exposure-mediated social deficits in mouse offspring.

### Effect of Prenatal Progestin Exposure

Our *in vitro* study in hypothalamic neurons showed that transient progestin treatment induces persistent epigenetic modifications even after removal of progestin and subsequently dissociates both ERβ and RORA from the OXT promoter, triggering OXT suppression. The *in vivo* study in mouse models showed that prenatal MPA exposure induces OXT suppression, partly contributing to social deficits in mouse models. In addition, the regular MPA dose for treatment of women contraception is reported as 150mg (49), and the high MPA dose for tumor suppression is in the range of 400-2000mg daily (50), and those doses can be calculated as 2.5-33.3mg/kg body weight if the average weight of women is considered as 60kg. Given the fact that the practical human exposure time can be 3 months (first trimester is most sensitive for ASD development) or more during the pregnancy (10, 51), while the exposure time of pregnant dams is much less, can only reach to 21 days in maximum, we finally chose MPA dose of 20mg/kg body weight for prenatal treatment of pregnant dams to mimic the possible high dose of MPA for human exposure. Furthermore, our *in vitro* and *in vivo* study showed that progestin exposure induces suppression of ERβ and RORA in addition to OXT suppression, which is consistent with our previous finding in rat models (7, 8), indicating that ERβ may play an important role in prenatal progestin exposure-mediated social deficits in mouse offspring. In addition, our results showed that prenatal progestin exposure triggers social deficits in rodents, which is consistent with previous reports that maternal hormone exposure is a potential risk factor for ASD (52, 53), modulating a neurogenic response and social recognition during development (54, 55).

### Role of ERβ and RORA in OXT Expression

Our *in vitro* study showed that the OXT promoter has the potential binding sites of ERβ and RORA, which are responsible for progestin treatment-mediated OXT suppression. This indicates that RORA may also play a role in OXT expression that adds to the significant effect of ERβ, which is consistent with previous findings that RORA plays a critical role in embryo development (35) and is associated with autism development (56). Interestingly, our *in vitro* study found that ERβ expression completely, while RORA expression partly, reverses progestin treatment-mediated OXT suppression. Furthermore, *in vivo* mouse study showed that postnatal ERβ expression completely, while RORA expression partly, reverses prenatal MPA-mediated OXT suppression in hypothalamic neurons. Postnatal ERβ expression can partly ameliorate prenatal MPA exposure-mediated social deficits in mouse offspring, while postnatal RORA expression has no effect. The results indicate that the effect of ERβ expression overcomes the effect of RORA expression, which can be explained with the hypothesis that ERβ expression-mediated SOD2 up-regulation (25) diminishes progestin exposure-mediated oxidative stress and epigenetic modifications (26), subsequently restoring the binding ability of both ERβ and RORA on the OXT promoter. In addition, it has been previously reported that OXT expression is regulated by estrogen and ERβ (57, 58). Sharma et al. has shown that ERβ forms a functional complex with cAMP response element-binding protein (CBP) and steroid receptor coactivator-1 (SRC1) in the presence of ERβ ligand, and subsequently regulating the OXT expression through ERE binding site on the OXT promoter (57). On the other hand, our results show that MPA treatment induces histone modification on the OXT promoter, resulting in ERβ dissociation from ERE binding motif on the OXT promoter, triggering OXT suppression. Furthermore, the progestin-responsive ERE binding motif identified in this work is different with previous study (57), and the progestin exposure-mediated OXT suppression is epigenetic modification-based persistent suppression. In this study, a novel mechanism for progestin-mediated OXT suppression through ERβ and RORA is reported.

### Role of OXT and Social Deficits

OXTR is expressed in a variety of human tissues and is highly expressed in limbic regions such as the amygdala (12, 20). It has been reported that the OXT/OXTR signaling pathway plays a role in regulation of a variety of social behaviors (11, 16) as well as ASD etiology (18, 19, 59) and is involved with anxiety-like behaviors (13, 14). Our results showed that OXTR expression does not change in response to progestin treatment, while OXT expression is reduced persistently. Furthermore, prenatal OXT deficiency in OXT knockdown mice partly mimics prenatal MPA exposure-mediated social deficits, including impaired social interaction and social ability, but showed no effect on anxiety-like behaviors, as measured in MBT and EPM tests. Furthermore, postnatal expression of ERβ in the PVN area or through postnatal OXT peptide injection in the third ventricle partly ameliorates prenatal MPA-exposure-mediated social deficits; again, there is no effect on anxiety-like behaviors. This can be partly explained through the hypothesis that postnatal OXT manipulation is only effective in certain OXT-responsive areas, but cannot mimic the whole endogenous OXT-responsive area (60). However, it is clear that OXT peptides do have some effect on modulating social behaviors in mouse offspring. On the other hand, recent placebo-controlled trial using intranasal OXT therapy showed no significant effect on ASD children and adolescents, which can be explained because intranasal OXT administration may not reach sufficient OXT concentrations in OXT-responsive areas of the central nervous system (61).

## Conclusions

Transient progestin treatment induces epigenetic changes, triggering persistent OXT suppression. Postnatal ERβ expression in hypothalamic regions or postnatal OXT peptide injection partly ameliorates postnatal MPA exposure-mediated impaired social interaction and social abilities in mouse offspring. We conclude that maternal progestin exposure-mediated oxytocin suppression contributes to social deficits in mouse offspring.

## Data Availability Statement

The original contributions presented in the study are included in the article/**Supplementary Material**. Further inquiries can be directed to the corresponding authors.

## Ethics Statement

The animal study was reviewed and approved by The Institutional Animal Care and Use Committee from Foshan Maternity & Child Healthcare Hospital at Southern Medical University.

## Author Contributions

PY wrote the paper. PY and XS designed, analyzed the data and interpreted the experiments. RS and HY performed part of the gene analysis. HZ performed part of the mouse experiments. SH and JZ performed the remaining experiments. All authors read and approved the final manuscript.

## Funding

This study was financially supported by Guangdong Basic and Applied Basic Research Foundation #: 2020A1515110644, Foshan Science and Technology Advanced Project #: 1920001000541 and The Social-Area Science and Technology Research Program of Foshan Project #: 2120001008276.

## Conflict of Interest

The authors declare that the research was conducted in the absence of any commercial or financial relationships that could be construed as a potential conflict of interest.

## Publisher’s Note

All claims expressed in this article are solely those of the authors and do not necessarily represent those of their affiliated organizations, or those of the publisher, the editors and the reviewers. Any product that may be evaluated in this article, or claim that may be made by its manufacturer, is not guaranteed or endorsed by the publisher.

## Abbreviations

ASD, autism spectrum disorders; ChIP, chromatin immunoprecipitation; EPM, elevated plus maze; ERE, estrogen response element; ERβ, estrogen receptor β; MBT, marble-burying test; MPA, medroxyprogesterone acetate; O2, superoxide anions; OXT, oxytocin; OXTR, oxytocin receptor; PVN, paraventricular nuclei; RORA, retinoic acid-related orphan receptor alpha; ROS, reactive oxygen species; SI, social interaction; SOD2, superoxide dismutase 2; USV, ultrasonic vocalization.
