# Supplementary material for: Prenatal Progestin Exposure-Mediated Oxytocin Suppression Contributes to Social Deficits in Mouse Offspring
Source: Front Endocrinol (Lausanne). 2022 Mar 15;13:840398. doi: 10.3389/fendo.2022.840398 (PMC8964973; doi:10.3389/fendo.2022.840398)
Supplement: Supplementary file 1 [file DataSheet_1.pdf]

Prenatal Progestin Exposure-Mediated Oxytocin Suppression Contributes to Social Deficits in Mouse Offspring

Saijun Huang[1,*], Jiaying Zeng[1,*], Ruoyu Sun[1], Hong Yu[1], Haimou Zhang[2], Xi Su[1,#], Paul Yao[1,#]

Supplemental Information

Data S1. MATERIALS AND METHODS

A detailed description can be found in Supplementary Information (see Data S1), and the related primers used in this study were shown in Table S1.

Reagents and materials. The primary hypothalamus neurons from the paraventricular nucleus (PVN) area of mice were isolated and cultured in DMEM medium plus 10% fetal bovine serum (FBS), 10% heat-inactivated defined horse serum, 20 mM D-glucose and 100 U/ml Pen/Strep (from Invitrogen). All cells were maintained in a humidified incubator with 5% CO_2 at 37°C. In some experiments, the cells were conditionally immortalized using an hTERT lentivirus vector with an extended life span to achieve higher transfection efficiency and experimental stability (1, 2).

The antibodies for β-actin (sc-47778), p53 (sc-126), RORA (sc-518081), RXRα (sc-515929) and SOD2 (sc-30080) were obtained from Santa Cruz Biotechnology. Antibody for 8-oxo-dG (4354-MC-050) was purchased from Novus Biologicals, while antibodies for acetyl-histone H4 K5, K8, K12, and K16 (H4K5,8,12,16ac, #PA5-40084) were obtained from Invitrogen. Antibodies for ERβ (ab3576), anti-histone H3 acetyl K9, K14, K18, K23, K27(H3K9,14,18,23,27ac, ab47915), H4K20me1 (ab9051), H4K20me3 (ab9053), H4R3me1 (ab17339), H3K9me2 (ab1220), H3K9me3 (ab8898), H3K27me2 (ab24684) and H3K27me3 (ab6002) were obtained from Abcam. 3-nitrotyrosine (3-NT) was measured using the 3-Nitrotyrosine ELISA Kit (ab116691 from Abcam), oxytocin (OXT) from tissue, culture medium, serum and cerebrospinal fluid (CSF) was determined using the Oxytocin ELISA Kit (ab133050) and 8-hydroxy-2' -deoxyguanosine (8-OHdG) formation was measured using an OxiSelect™ Oxidative DNA Damage ELISA Kit (Cat No. STA320, from Cell Biolabs Inc.) according to manufacturers' instructions. The mitochondrial fraction was isolated using a Pierce Mitochondria Isolation Kit (Pierce Biotechnology) per manufacturers' instructions. Protein concentration was measured using the Coomassie Protein Assay Kit (Pierce Biotechnology). Luciferase activity assay was carried out using the Dual-Luciferase™ Assay System (Promega) and the transfection efficiency was normalized using a cotransfected renilla plasmid (3).

17β-estradiol (E2, #E2758); progesterone (P4, #P0130); levonorgestrel (LNG, #1362602); medroxyprogesterone acetate (MPA, #1378001); nestorone (NES, # SML0550); norethindrone (NET, #1469005); norethindrone acetate (NETA, #1470004); norgestimate (NGM, # 1471914); hydroxyprogesterone caproate (OHPC, #1329006) and oxytocin acetate salt hydrate (#06379) were obtained from Sigma. Norethynodrel (NEN, #E4600-000) was obtained from Steraloids.

RT reaction and real-time quantitative PCR. Total RNA from treated cells was extracted using the RNeasy Micro Kit (Qiagen) and the RNA was reverse transcribed using an Omniscript RT kit (Qiagen). All the primers were designed and verified by agarose gel, and the details are provided in supplementary Table 1. Real-time quantitative PCR was run on iCycler iQ (Bio-Rad) with the Quantitect SYBR green PCR kit (Qiagen). PCR was performed by denaturing at 95°C for 8 min

followed by 45 cycles of denaturation at 95°C, annealing at 60°C, and extension at 72°C for 10s, respectively. β-actin was used as the housekeeping gene for transcript normalization, and the mean values were used to calculate relative transcript levels with the $^{\Delta\Delta}$CT method per instructions from Qiagen.

Western blotting. Treated cells were lysed in an ice-cold lysis buffer with the addition of protease inhibitor cocktail (Sigma), and the protein concentration was measured using the Coomassie Protein Assay Kit (Pierce Biotechnology). Certain amounts of proteins (20-35g) were loaded and separated in 10% SDS-PAGE gel before the proteins were then transferred to a PVDF membrane. The membrane was blotted by primary antibodies to be left overnight and subsequently incubated with differentially labeled species-specific secondary antibody using either anti-RABBIT IRDye™ 800CW (green) or anti-MOUSE (or goat) ALEXA680 (red) for 2 hours. After thorough washing, the membranes were scanned and quantitated using the ODYSSEY Infrared Imaging System (LI-COR, NE).

Luciferase reporter assay. Cells were seeded in a 6-well plate and cultured in complete medium until 80% confluent. Cells were then co-transfected by 3 µg of related reporter plasmid as well as 0.2 µg of pRL-CMV-Luc *Renilla* plasmid (from Promega) for internal transfection efficiency control. 24 hours after transfection, cells were further treated as indicated and then harvested. Luciferase activity were determined using Dual-Luciferase^{TM} Assay System (Promega) as per manufacturers' instructions. The transfection efficiencies were calculated accordingly using the *Renilla* plasmid and the reporter activities were normalized and calculated.

Chromatin immunoprecipitation (ChIP). Cells were treated by 1% formaldehyde for 20 min for crosslinking and then terminated by adding 0.1 M of glycine. Cells were scraped from plates and sonicated following by brief centrifugation, and the subsequent protein supernatant was used for determination of protein concentrations using the Coomassie Protein Assay Kit (Pierce Biotechnology). 500 µg of protein solution were incubated with BSA/salmon sperm DNA, preimmune IgG, and a slurry of Protein A Agarose beads for pre-clearing. The immunoprecipitations were then performed by adding the indicated antibodies, BSA/salmon sperm DNA and a 50% slurry of Protein A agarose beads to be left overnight. The related inputs and immunoprecipitates were thoroughly washed, eluted, and then incubated with 0.2 mg/ml of Proteinase K at 42˚C for 2 hours. They were then switched to 65°C for another 6 hours to reverse crosslinking. DNA fragments were extracted by phenol/chloroform followed by ethanol precipitation and the subsequent DNA solution was used for real time PCR (qPCR). An ~150 bp fragment on the related promoters was then amplified using the primers provided in supplementary Table 1.

Measurement of oxidative stress. The intracellular ROS formation was measured by CM-H2DCFDA-based fluorescence emission. In brief, treated cells were plated in a 24-well plate and cultured until 80% confluent. 10 µM of CM-H2DCFDA (Invitrogen) were then added and incubated for 45 min at 37°C, and ROS formation was determined at 485/530nm of excitation/emission wavelength by using the FLx800 microplate fluorescence reader (Bio-Tek). The results were normalized using a series of H2O2 as standard (3, 4). 8-OHdG formation was measured using an OxiSelect™ Oxidative DNA Damage ELISA Kit (Cat No. STA320, from Cell Biolabs Inc.) per manufacturers' instructions and the formation of 8-oxo-dG was determined by immunostaining and quantitated by Image J.

SOD2 activity assay. SOD2 was obtained from the mitochondrial fraction that was isolated using a Pierce Mitochondria Isolation Kit (Pierce) according to manufacturers' instructions. SOD activity was measured as described previously (5). In brief, a stable O2.- source was generated through

the conversion action of XOD (xanthine oxidase) from xanthine and mixed with chemiluminescent (CL) reagents to achieve a stable light emission. The SOD2 sample injection can scavenge O2.- and the subsequent decrease of chemiluminescent response is proportional to SOD2 activity. This system can have a detection limit of 0.001 U/ml with the linear range of 0.03~2.00 U/ml. The results were normalized by protein concentration and expressed as Units/mg proteins (U/mg) (6).

Immunostaining. Treated cells were transferred and incubated on cover slips. Cells were then washed by PBS and fixed by 4% of paraformaldehyde for 20 min followed by incubation with 0.3% Triton X-100 in PBS for 15 min. The cells were blocked by normal goat serum and then incubated with the 8-oxo-dG anti-mouse antibody (#4354-MC-050, from Novus Biologicals) at 4°C to be left overnight. They were subsequently incubated with secondary antibody Alexa Fluor 488 for 2 hours. The cover slips were then mounted by antifade Mountant with DAPI (for nuclei staining, in blue). The representative pictures were taken using a confocal laser microscope and the staining was further quantitated by Image J. software.

In vivo mouse experiments. The animal protocol conformed to US NIH guidelines (Guide for the Care and Use of Laboratory Animals, No. 85-23, revised 1996) and was reviewed and approved by the Institutional Animal Care and Use Committee from Foshan Maternity and Child Health Care Hospital. Adult (3-month-old) female mice with either WT or $OXT^{-/-}$ backgrounds were monitored for estrous cycles with daily vaginal smears. Only mice with at least two regular 4- to 5-day estrous cycles were included in the studies.

Animal behavior test.
Marble-burying test (MBT). The experimental mouse was placed in a clean cage (35×23×19 cm^3) that was filled with wood chip bedding (5 cm depth) containing 20 of colored 1 cm-diameter glass marbles with a 5×4 arrangement. The number of buried marbles (>50% covered by bedding material) was counted in 30 minutes by a qualified double-blinded technician (7-10).

Elevated plus maze (EPM). The EPM test was performed to characterize anxiety-like behavior in mouse offspring. The Elevated Plus Maze Package together with IR Beam Detection (Cat #: MED-ELVM-1R) was purchased from Med Associates Inc. The maze has two sets of open and closed arms, and dual sensors located at the entrance to each goal runway can differentiate between runway exploration and entrance for more accurate position detection. The experimental mouse was placed in the junction area. Movements were quantitated by infrared beams installed on each arm for 5 min and then automatically recorded by MED-PC software (Cat #: SOF-735, Med Associates). The time spent in both the open and closed arms was quantitated by a double-blinded technician. In addition, total motor activity was counted in an open-field test during a 30 min assessment (9, 11).

Ultrasonic vocalizations (USV). The USV test was conducted on postnatal day 7 during a maternal-separation paradigm. The individual pup was isolated and placed in a sound-proof chambers. The USV were recorded by an externally polarized condenser microphone (30-300 kHz) that was attached 15-20 cm above the floor of the isolation chamber and the microphone was connected to Avisoft-UltrasoundGate recording software (from Avisoft Bioacoustics) before the pup-emitted calls were transferred to WAV sound files. Recorded number of USV was analyzed by a generalized linear model through a negative binomial distribution and a log-link function (12, 13).

Social interaction (SI) test. The social interaction test was performed in an open-field arena using the Open Field Starter Package for Mouse (Cat#: MED-OFAS-MSU, Med Associates Inc.). The subjects, including Test and Stranger mice, were habituated in the arena separately for 5 min

before the test. During the test, the mice were placed into the apparatus for 20 min and the time spent following, mounting, grooming, and sniffing any body parts of the other mouse was counted as an indicator of social engagement. The social interaction time was quantitated and analyzed using EthoVision XT animal tracking software (14). Each "Stranger" mouse during the test was used only once, had no previous contact with the test mouse, and was matched on the basis of sex, weight, and age (7-10).

Three-chambered social test. 7-8 week old mice were employed for measurement of sociability and preference for social novelty. Target subjects, including Stranger 1 and Stranger 2, were placed inside the wire cages for 3 days before the beginning of testing, and the test mice were placed in the testing room for at least 45 min before the start of behavioral tests. For the sociability test, the test mouse was placed in the middle chamber and left to habituate for 5 min, after which an unfamiliar Stranger 1 mouse was introduced into a wire cage in one of the side-chambers and an empty wire cage on the other side-chamber. The test mouse was allowed to explore all 3 chambers freely for 10 min. Following this, a novel Stranger 2 mouse was introduced into the previously empty wire cage and the test mouse was again left to explore freely for 10 min. All the parameters, including time spent in each chamber, number of entries into the chambers and track maps, were recorded and calculated by automated SMART software (15, 16).

In vivo superoxide anion ($O_2^{\cdot-}$) release. Superoxide anion release in amygdala tissues were extracted by dimethyl sulfoxide-tetrabutylammonium chloride (DMSO-TBAC) solution and the TBAC-$O_2^{\cdot-}$ complex was then further detected by use of the luminol-EDTA-Fe enhanced chemiluminescence system (5). In brief, the biological tissues were isolated and purged continuously by N2 gas to remove traces of oxygen. $O_2^{\cdot-}$ from tissues was extracted by DMSO-TBAC and then the chemiluminescent reagents and $O_2^{\cdot-}$ extract solutions were pumped into a glass scintillation vial, which was placed in the luminometer. The chemiluminescence intensity was continuously monitored for 2 min and calculated. Superoxide levels were calculated from the standard curve generated by the xanthine/xanthine oxidase reaction (4).

Statistical analysis. The data was given as mean ± SD and all the experiments were performed at least in quadruplicate unless indicated otherwise. The n=4 or 5 was used for measurements of biological parameters, and n=9 was used for the analysis of animal behaviors. One-way analysis of variance (ANOVA) followed by the Turkey−Kramer test was used to determine statistical significance of different groups, and the two-way ANOVA followed by the Bonferroni post hoc test was used to determine the differences of two factors (e.g. OXT deficiency and MPA treatment) using SPSS 22 software, with a P value of < 0.05 being considered significant.

REFERENCES

1. Bodnar AG, Ouellette M, Frolkis M, Holt SE, Chiu CP, Morin GB, et al. Extension of life-span by introduction of telomerase into normal human cells. *Science.* 1998;279(5349):349-52.
2. Kong D, Zhan Y, Liu Z, Ding T, Li M, Yu H, et al. SIRT1-mediated ERbeta suppression in the endothelium contributes to vascular aging. *Aging Cell.* 2016;15(6):1092-102.
3. Zhang H, Li L, Li M, Huang X, Xie W, Xiang W, et al. Combination of betulinic acid and chidamide inhibits acute myeloid leukemia by suppression of the HIF1alpha pathway and generation of reactive oxygen species. *Oncotarget.* 2017;8(55):94743-58.
4. Yao D, Shi W, Gou Y, Zhou X, Yee Aw T, Zhou Y, et al. Fatty acid-mediated intracellular iron translocation: a synergistic mechanism of oxidative injury. *Free Radic Biol Med.* 2005;39(10):1385-98.

5. Yao D, Vlessidis AG, Gou Y, Zhou X, Zhou Y, and Evmiridis NP. Chemiluminescence detection of superoxide anion release and superoxide dismutase activity: modulation effect of Pulsatilla chinensis. *Anal Bioanal Chem.* 2004;379(1):171-7.
6. Kong D, Zhan Y, Liu Z, Ding T, Li M, Yu H, et al. SIRT1-mediated ERbeta suppression in the endothelium contributes to vascular aging. *Aging Cell.* 2016.
7. Bahi A. Sustained lentiviral-mediated overexpression of microRNA124a in the dentate gyrus exacerbates anxiety- and autism-like behaviors associated with neonatal isolation in rats. *Behav Brain Res.* 2016;311:298-308.
8. Bahi A. Hippocampal BDNF overexpression or microR124a silencing reduces anxiety- and autism-like behaviors in rats. *Behav Brain Res.* 2017;326:281-90.
9. Zou Y, Lu Q, Zheng D, Chu Z, Liu Z, Chen H, et al. Prenatal levonorgestrel exposure induces autism-like behavior in offspring through ERβ suppression in the amygdala. *Mol Autism.* 2017;8:46.
10. Xie W, Ge X, Li L, Yao A, Wang X, Li M, et al. Resveratrol ameliorates prenatal progestin exposure-induced autism-like behavior through ERβ activation. *Mol Autism.* 2018;9:43.
11. Hu M, Richard JE, Maliqueo M, Kokosar M, Fornes R, Benrick A, et al. Maternal testosterone exposure increases anxiety-like behavior and impacts the limbic system in the offspring. *Proc Natl Acad Sci U S A.* 2015;112(46):14348-53.
12. Silverman JL, Yang M, Lord C, and Crawley JN. Behavioural phenotyping assays for mouse models of autism. *Nat Rev Neurosci.* 2010;11(7):490-502.
13. Schaafsma SM, Gagnidze K, Reyes A, Norstedt N, Mansson K, Francis K, et al. Sex-specific gene-environment interactions underlying ASD-like behaviors. *Proc Natl Acad Sci U S A.* 2017;114(6):1383-8.
14. Mufford JT, Paetkau MJ, Flood NJ, Regev-Shoshani G, Miller CC, and Church JS. The development of a non-invasive behavioral model of thermal heat stress in laboratory mice (Mus musculus). *J Neurosci Methods.* 2016;268:189-95.
15. Moy SS, Nadler JJ, Perez A, Barbaro RP, Johns JM, Magnuson TR, et al. Sociability and preference for social novelty in five inbred strains: an approach to assess autistic-like behavior in mice. *Genes Brain Behav.* 2004;3(5):287-302.
16. Wang X, Lu J, Xie W, Lu X, Liang Y, Li M, et al. Maternal diabetes induces autism-like behavior by hyperglycemia-mediated persistent oxidative stress and suppression of superoxide dismutase 2. *Proc Natl Acad Sci U S A.* 2019;116(47):23743-52.

Table S1. Sequences of primers for the real time quantitative PCR (qPCR)

Gene	Species	Analysis	Forward primer (5'→3')	Reverse primer (5'→3')
OXT	Mouse	ChIP	cttccaggctgcttctcttt	gacagcatgactggtcacaga
β-actin	Mouse	mRNA	tcttgggtatggaatcctgtg	atctccttctgcatcctgtca
ERβ	Mouse	mRNA	atgtgctatggccaacttctg	caagcttcctcttcagggtct
OXT	Mouse	mRNA	acctggatatgcgcaagtgt	cgaaggcaggtagttctcctc
OXTR	Mouse	mRNA	caacccatggatctacatgct	gactcaggacgaaggtggag
RORA	Mouse	mRNA	attggacatcaatgggatcaa	tttggatatgttctgggcaag
SOD2	Mouse	mRNA	ggcctacgtgaacaatctcaa	tcaggtttgtccagaaaatgg
SYP	Mouse	mRNA	ttcgctttcatgtggctagtt	aagtcacagggtccctcagtt
loxP	Mouse	Genotype	ttgtgtataccaccacaagtgcacc	agtacaggacacttcggtgtctacc
Cre	Mouse	Genotype	cttgggctgccagaatttctc	cccagaaatgccagattacg

FIGURE S1

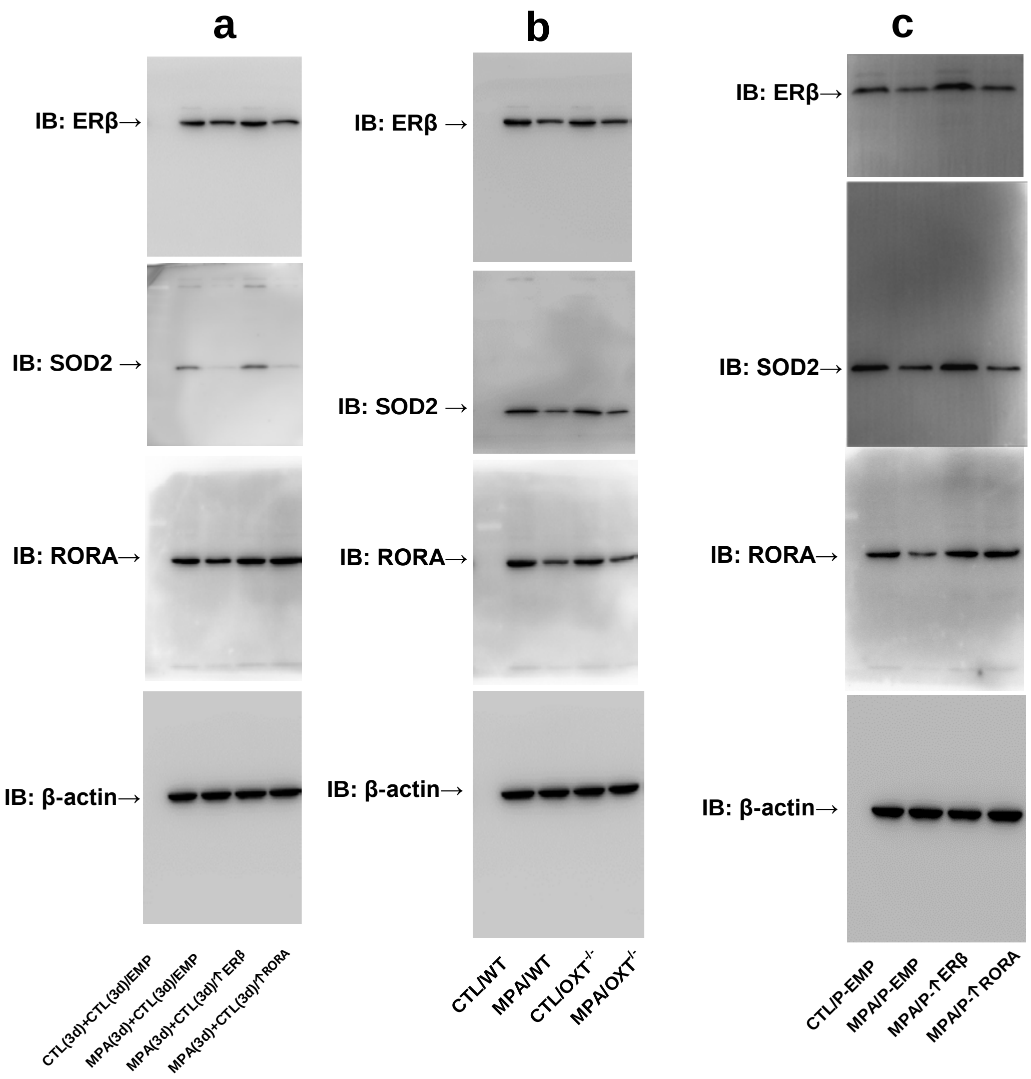

Fig S1. Representative pictures of full blots for Western Blotting. (a) Full blots for Figure 1d. (b) Full blots for Figure 3c. (c). Full blots for Figure 5c.

FIGURE S2

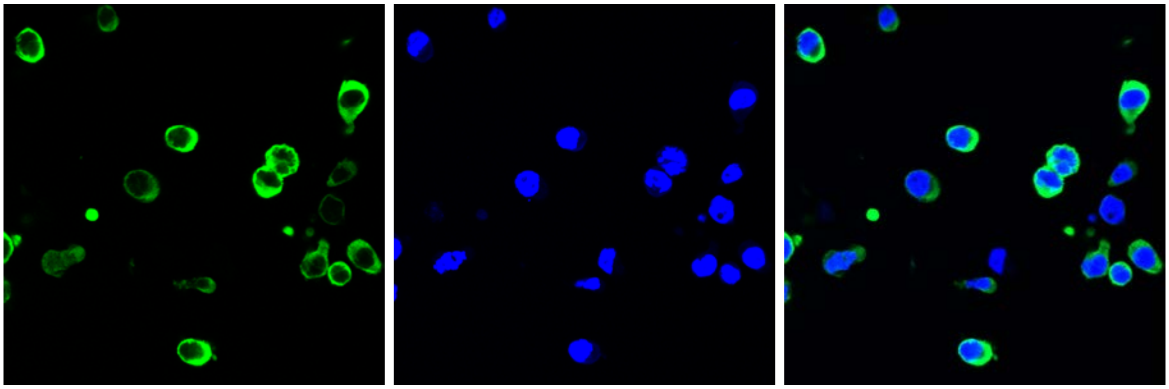

Fig S2. Isolation and characterization of OXT expression neurons. Primary mouse hypothalamic neurons were isolated from hypothalamic PVN area for immunostaining using OXT antibody (green) and DAPI staining for nuclei (blue).

FIGURE S3

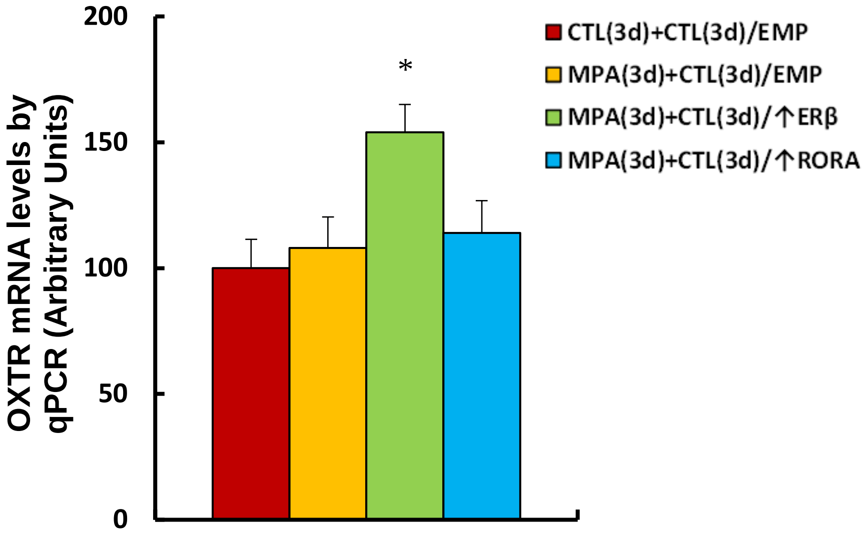

Fig S3. Transient MPA treatment causes no change, while ERβ expression significantly increases OXTR expression. Mouse hypothalamic neurons were treated with either 10μM MPA or vehicle control (CTL) for 3 days, the cells were then infected by empty (CTL), ERβ expression (↑ERβ), or RORA expression (↑RORA) lentivirus on day 3 and the cells were then cultured without MPA for another 3 days in the presence of 1% serum before being harvested for analysis of OXTR mRNA levels, n=4. *, $P<0.05$, vs. CTL(3d)+CTL(3d)/EMP group. Data are expressed as mean ± SD.

FIGURE S4

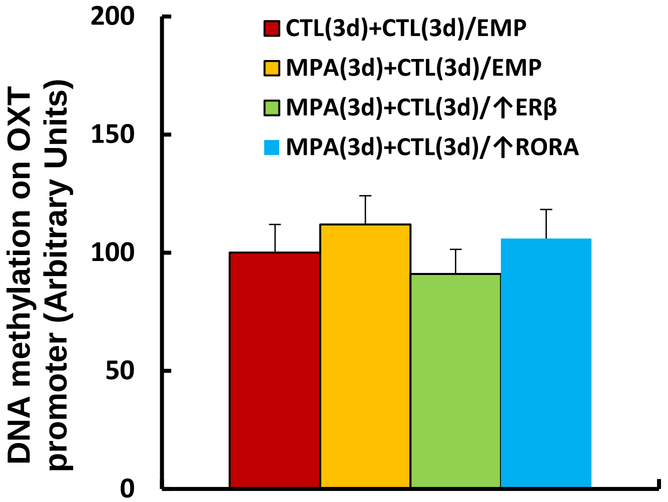

Fig S4. Potential effect of MPA and ERβ expression on the DNA methylation on the OXT promoter. Mouse hypothalamic neurons were treated with either 10μM MPA or vehicle control (CTL) for 3 days, the cells were then infected by empty (CTL), ERβ expression (↑ERβ), or RORA expression (↑RORA) lentivirus on day 3 and the cells were then cultured without MPA for another 3 days in the presence of 1% serum before being harvested for analysis of DNA methylation on the OXT promoter, n=4. Data are expressed as mean ± SD.

FIGURE S5

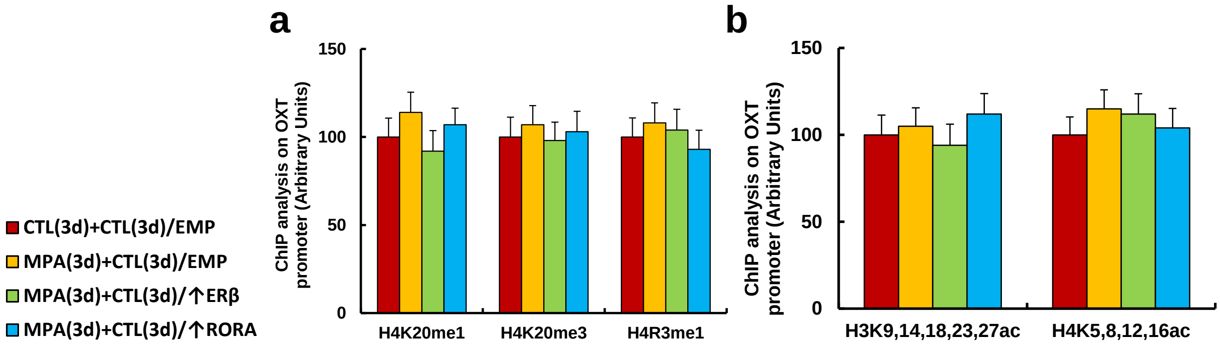

Fig S5. Potential effect of MPA and ERβ expression on the epigenetic changes on the OXT promoter. Mouse hypothalamic neurons were treated with either 10μM MPA or vehicle control (CTL) for 3 days, the cells were then infected by empty (CTL), ERβ expression (↑ERβ), or RORA expression (↑RORA) lentivirus on day 3 and the cells were then cultured without MPA for another 3 days in the presence of 1% serum before being harvested for ChIP analysis. (a) Histone H4 methylation on the OXT promoter, n=4. (b) Histone acetylation on the OXT promoter using H3K9,14,18,23,27ac and H4K5,8,12,16ac antibodies, n=4. Data are expressed as mean ± SD.

FIGURE S6

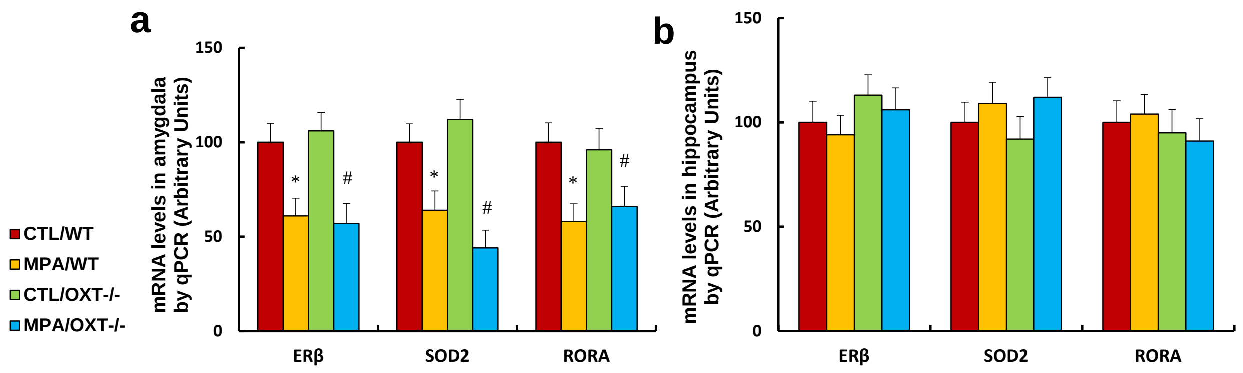

Fig S6. Potential effect of Prenatal OXT deficiency on prenatal MPA exposure-mediated gene expression in offspring. The OXT wild type (WT) or OXT null ($OXT^{-/-}$) background dams were exposed by either control (CTL) or MPA, and the brain tissues from subsequent offspring were isolated for mRNA analysis. (a) mRNA levels in amygdala, n=4. (b) mRNA levels in hippocampus, n=4. *, $P<0.05$, vs CTL/WT group; #, $P<0.05$, vs CTL/OXT-/- group. Data are expressed as mean ± SD.

FIGURE S7

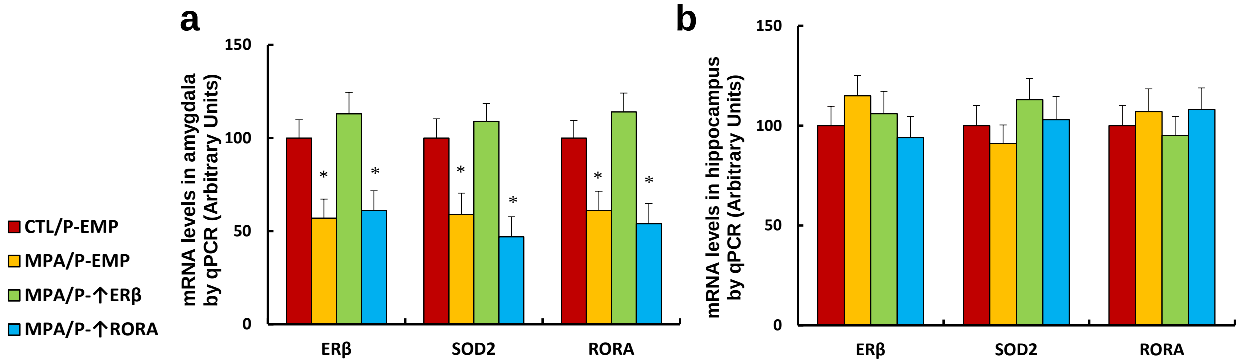

Fig S7. Potential effect of postnatal ERβ expression on prenatal MPA exposure-mediated gene expression in offspring. The pregnant dams were treated by either control (CTL) or MPA, and the subsequent male offspring received either empty (EMP), ERβ (↑ERβ) or RORA (↑RORA) lentivirus in MBH area, then the animals were sacrificed, and the brain tissues from subsequent offspring were isolated for mRNA analysis. (a) mRNA levels in amygdala, n=4. (b) mRNA levels in hippocampus, n=4. *, P<0.05, vs CTL/P-EMP group. Data are expressed as mean ± SD.
